# Graft-Free Sinus Lift Using a Resorbable Magnesium Membrane vs. Standard Grafting Protocol: A Bilateral Case Report with Histological Evaluation

**DOI:** 10.3390/dj14060381

**Published:** 2026-06-18

**Authors:** Cristian Scognamiglio, Alessandro Perucchi, Marija Čandrlić, Željka Perić Kačarević

**Affiliations:** 1 Private Practice, Studio Odontoiatrico Dr. Perucchi, Via Luigi Lavizzari 20, 6850 Mendrisio, Switzerland; c.scognamiglio93@gmail.com (C.S.); alessandroperucchi@sunrise.ch (A.P.); 2 Latin American Institute of Dental Research and Education—ILAPEO, Curitiba 80710-150, PR, Brazil; 3 Department of Dental Medicine, Faculty of Dental Medicine and Health Osijek, J.J. Strossmayer University of Osijek, Crkvena 21, 31000 Osijek, Croatia; marija.candrlic@fdmz.hr; 4 Department of Anatomy, Histology, Embriology, Pathology Anatomy and Pathology Histology, Faculty of Dental Medicine and Health Osijek, J.J. Strossmayer University of Osijek, Crkvena 21, 31000 Osijek, Croatia

**Keywords:** sinus lift, guided tissue regeneration, magnesium, collagen, histology

## Abstract

**Objective**: To present a split-mouth bilateral case report comparing a graft-free sinus lift technique developed by C.S. and A.P. using a resorbable magnesium membrane with a standard lateral sinus augmentation using xenograft and collagen membrane. The two approaches were evaluated through clinical, radiographic, and histological outcomes. **Methods**: A healthy 68-year-old female patient underwent bilateral sinus augmentation in the posterior maxilla. On the right side, a standard sinus lift was performed using a xenogeneic bone graft in combination with a resorbable collagen membrane. On the left side, a graft-free approach was done using only a resorbable magnesium membrane to support the elevated sinus membrane. **Results:** After a six-month healing period, implants were placed, and bone core biopsies were harvested for histological evaluation. Histological analysis revealed 56.4% newly formed bone in the graft-free magnesium membrane site and 22.5% in the grafted control site. A two-year radiological follow-up confirmed complete bone maturation and implant stability on both sides. **Conclusions**: This case promotes the potential of a graft-free sinus lift approach using a resorbable magnesium membrane as a promising alternative to conventional grafting protocols. The beneficial clinical, radiological and histological outcomes suggest that this technique may simplify the sinus augmentation procedure and should be further explored in larger clinical studies.

## 1. Introduction

Restoration of the edentulous posterior maxilla with dental implants is often a significant clinical challenge due to the reduced vertical bone height and bone volume. This condition is primarily the result of post-extraction alveolar bone resorption and maxillary sinus pneumatization, both of which limit the available bone for implant placement [1,2]. The maxillary sinus is the largest of the paranasal sinuses, pyramidal in shape, and lined by a mucoperiosteal membrane known as the Schneiderian membrane. Histologically, this membrane is bilaminar, consisting of ciliated columnar epithelium on its internal side and periosteum on its osseous side. The sinus varies significantly in size among individuals and even between the right and left sides of the same skull [3].

To overcome the reduction in vertical bone height, maxillary sinus floor elevation (sinus lift) has become a widely accepted surgical procedure in implant dentistry. Two primary techniques are used for maxillary sinus augmentation. The lateral (open) approach provides direct access to the sinus cavity and is indicated for severely atrophic ridges, whereas the transcrestal (closed) approach is less invasive and suitable for cases with moderate residual bone height [4,5]. Both techniques aim to elevate the Schneiderian membrane and promote new bone formation in the augmented sinus floor [6].

The most used biomaterials for bone regeneration in sinus lift procedures include autologous bone, allografts, bovine xenografts and alloplasts [7]. However, the traditional sinus grafting method has faced criticism recently and the necessity of bone grafts has been questioned. Since the elevated Schneiderian membrane itself possesses osteogenic potential, spontaneous bone regeneration can occur if the membrane is lifted and a stable blood clot is maintained in the subantral space [8,9]. In a recent systematic review and meta-analysis of randomized controlled trials, Lie et al. [10] critically evaluated the clinical evidence on graft-free maxillary sinus membrane elevation for implantation in the atrophic posterior maxilla. The results revealed high overall implant survival rates for both graft-free and grafted sinus lift groups (97.92% and 98.73%, respectively). However, the graft-free group showed a significantly lower vertical bone height gain and lower bone density. Beyond the selection of bone graft materials, the question of whether resorbable or non-resorbable membranes result in superior outcomes in sinus lift procedures has emerged. A study by Barone et al. [11] reported that the use of a membrane reduces the proliferation of connective tissue and the graft resorption rate. It is possible that the blood supply of the maxillary sinus may play a role in such outcomes.

In the context of guided bone regeneration, a recently developed resorbable magnesium membrane has shown excellent biological and mechanical performance. It provides high mechanical stability and space maintenance, demonstrates good biocompatibility, and undergoes gradual resorption accompanied by the release of magnesium ions [12]. Magnesium and its degradation products have been extensively studied for their beneficial effects on bone healing and regeneration. Magnesium ions stimulate osteoblast proliferation, improve alkaline phosphatase activity, and promote matrix mineralization, processes that are essential for early bone formation. Moreover, magnesium modulates the inflammatory environment and supports balanced bone remodeling, creating conditions favorable for bone regeneration [13,14,15,16,17,18,19].

Previous evidence on the clinical use of magnesium membranes in sinus augmentation is limited. A recently published case series by Elad et al. [20] demonstrated the successful application of a magnesium membrane for Schneiderian membrane repair during sinus lift procedures in four patients. In all cases, bone augmentation was performed using a mixture of xenograft and allograft material, and substantial vertical bone gains (10–20 mm) were observed within four months. Significantly, no residual magnesium membrane was detected upon clinical re-entry, and implant placement was successful in all cases. However, a graft-free approach was not adopted in this case series, and the magnesium membrane was solely used as a repair material rather than as part of a regenerative strategy.

Previously mentioned beneficial properties of the magnesium membrane support the hypothesis that combining the graft-free sinus lift concept with the magnesium membrane could create a protected and stable compartment beneath the elevated Schneiderian membrane, thereby supporting bone regeneration [12,21]. Recent clinical evidence supports the feasibility of graft-free sinus augmentation with high implant survival rates [10], while magnesium-based membranes have shown favorable mechanical stability and biocompatibility in sinus repair [20]. Therefore, we aimed to address this gap in the literature with the present bilateral case report. This is the first documented bilateral comparison evaluating a graft-free sinus lift performed with a resorbable magnesium membrane against a conventional xenograft + collagen membrane approach, supported by paired radiological and histological analyses.

## 2. Case Report

### 2.1. An Overview of the Materials and Methods Used in the Case Report

On one side of the posterior maxilla, a conventional sinus lift technique was performed using a xenogeneic bone graft (Straumann XenoGraft^®^, Basel, Switzerland) combined with a resorbable collagen membrane derived from procine pericardium (jason^®^ membrane, botiss biomaterials GmbH, Zossen, Germany). This material was selected in accordance with the surgeon’s routine clinical protocol for lateral sinus augmentation, due to its established osteoconductive properties and predictable long-term performance in maxillary sinus grafting [22]. On the contralateral side, a graft-free sinus lift was carried out using only a resorbable magnesium membrane (NOVAMag^®^ membrane, botiss biomaterials GmbH, Zossen, Germany).

**Figure 1:**
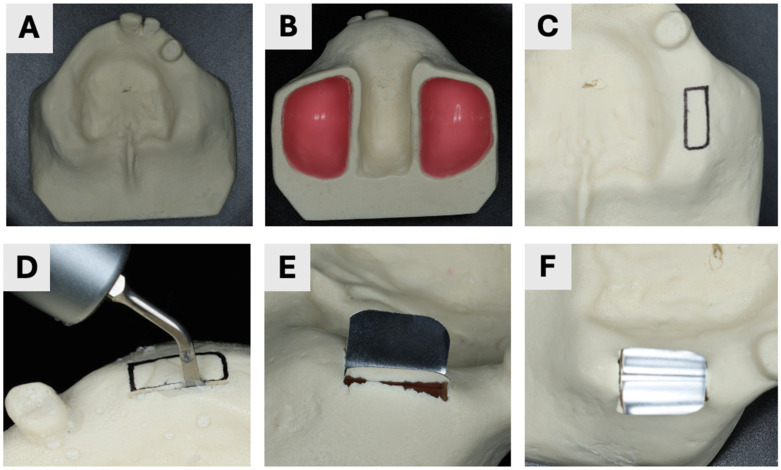
Preoperative use of an anatomical model for simulation and preshaping of the magnesium membrane. (**A**) Occlusal view of the maxillary anatomical model. (**B**) Superior view showing the bilateral maxillary sinuses. (**C**) Marking of the intended osteotomy site for the graft-free sinus lift on the left posterior maxilla. (**D**) Simulation of osteotomy preparation using a piezosurgical tip. (**E**) Insertion of the preformed resorbable magnesium membrane into the simulated sinus lift site. (**F**) Final adaptation of the magnesium membrane, showing a precise fit into the simulated sinus window.

The patient provided written informed consent in accordance with Swiss legal requirements (HRO Art. 2, Swiss Federal Act on Research Involving Human Beings). The retrospective analysis and histological evaluation were approved by the Institutional Ethics Committee of the Faculty of Dental Medicine and Health, J.J. Strossmayer University of Osijek, Croatia (Class: 602-01/23-12/05; No. 2158/97-97-10-23-03, approved on 24 January 2024). To prepare the osteotomy site and elevate the sinus membrane, we used the Piezomed system [W&H kit 0 (Zero) Bone Graft Sinus Lift Concept by C.S and A.P, Bürmoos, Austria] piezosurgical instrumentation. The piezosurgery tips used for membrane elevation and site preparation included: B6, B7, S2, S3 for initial and apical osteotomy preparation, and B2L and B2R for lateral refinement. Prior to surgery, the magnesium membrane was pre-shaped and tested on an anatomical model to assess its handling and adaptability to the surgical site (Figure 1).

### 2.2. Surgical Procedure of Bilateral Maxillary Sinus Lift and Provisional Prosthesis

The patient was a 68-year-old, non-smoking female with good oral hygiene and a long history of complete upper removable denture use. Her primary motivation for treatment was to replace the removable prosthesis with a fixed implant-supported restoration in the maxilla.

**Figure 2:**
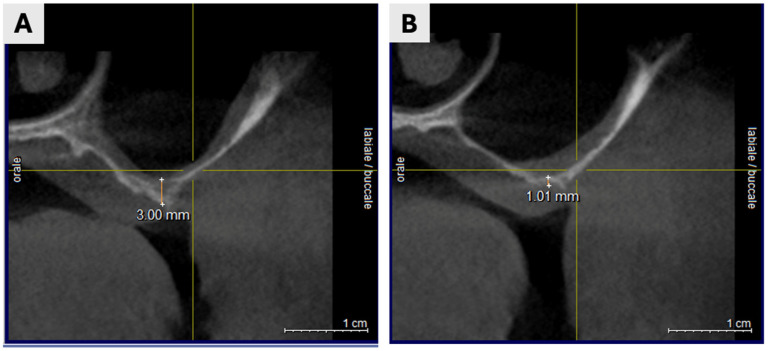
Pre-operative cone-beam computed tomography (CBCT) assessment of residual bone height in the posterior maxilla. (**A**) Right maxillary sinus showing a residual bone height of 3.00 mm. (**B**) Left maxillary sinus showing a residual bone height of 1.01 mm. No sinus pathology, septa, or Schneiderian membrane abnormalities were detected.

Radiological evaluation using cone-beam computed tomography (CBCT) revealed a severely atrophic posterior maxilla bilaterally. The residual bone height measured 1.01 mm in the left maxillary sinus and 3.00 mm in the right maxillary sinus, confirming the indication for sinus floor elevation on both sides. Both maxillary sinuses demonstrated similar anatomical features, including a regular sinus contour, absence of sinus septa, and intact Schneiderian membranes without signs of thickening or pathology (Figure 2).

The bilateral sinus lift procedure was performed collaboratively by two experienced clinicians (C.S. and A.P.). The patient received antibiotic prophylaxis with sulfamethoxazole/trimethoprim (NOPIL^®^ forte 800/160 mg, Mepha Pharma AG, Basel, Switzerland), administered orally twice daily (1-0-1) for five days, starting 24 h prior to surgery. Local anesthesia was administered using articaine with epinephrine (RUDOCAÏNE^®^ forte, Streuli Pharma AG, Basel, Switzerland). A total of 2 × 1.7 mL was administered in the first quadrant and 2 × 1.7 mL in the second quadrant.

**Figure 3:**
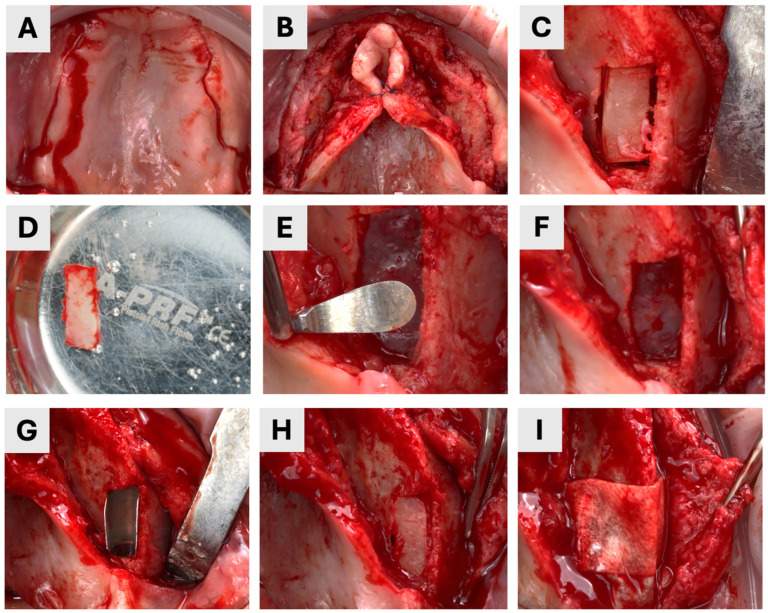
Step-by-step clinical procedure of a graft-free sinus lift performed on the patient’s left side using a resorbable magnesium membrane. (**A**) Initial mid-crestal incision extending along the alveolar ridge. (**B**) Full thickness mucoperiosteal flap elevation exposing the lateral wall of the maxilla. (**C**) Osteotomy preparation of the lateral sinus wall using piezosurgical instrumentation, following the previously described protocol. (**D**) The removed lateral bone window preserved in sterile saline during the procedure. (**E**) Gentle elevation of the Schneiderian membrane using custom-designed, flexible sinus elevation instruments. (**F**) View of the sinus cavity after successful membrane elevation. (**G**) Insertion of the pre-shaped magnesium membrane into the sinus cavity through the lateral window. (**H**) Replacement of the preserved bone window over the magnesium membrane. (**I**) Final coverage of the site with a resorbable porcine pericardium collagen membrane.

A full-thickness mucoperiosteal flap was elevated bilaterally. The osteotomy on the left side (graft-free side) was performed using piezosurgical tips (W&H Zero Bone Graft Sinus Lift Concept) as previously described in Section 2.1. A bone window was carefully removed and preserved in sterile saline. The sinus membrane was elevated using custom-designed instruments, Flexible Elevators Zero Bone Graft Sinus Lift Concept by Schwert, developed in collaboration with the company A. Schweickhardt GmbH u. Co. KG (Seitingen-Oberflacht, Germany). These included a set of four reusable, bendable sinus elevation tools that automatically regain their original shape after sterilization. Following elevation of the Schneiderian membrane, the resorbable magnesium membrane was carefully inserted through the crestal access. The preserved bone window was repositioned, and the site was covered with a resorbable collagen membrane without the use of any bone grafting material (Figure 3).

**Figure 4:**
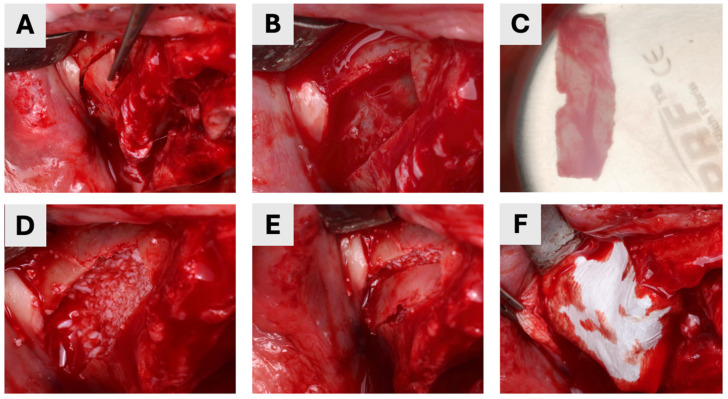
Step-by-step clinical procedure of a conventional lateral window sinus lift performed on the patient’s right side using xenogeneic bone grafting and a collagen membrane. (**A**) Access to the maxillary sinus through a lateral window approach using piezosurgical instrumentation (W&H 0 Bone Graft Sinus Lift Concept) and removing the bone window. (**B**) Reflection of the lateral bone window to expose the Schneiderian membrane. (**C**) Preservation of the bone window in sterile saline. (**D**) Placement of bovine-derived xenograft material into the elevated sinus cavity. (**E**) Repositioning of the original bone window. (**F**) Final coverage of the site with a resorbable porcine pericardium collagen membrane.

On the contralateral (right) side, a conventional lateral sinus lift was performed. The site was augmented using a xenogeneic bone substitute and covered with a resorbable collagen membrane (Figure 4).

**Figure 5:**
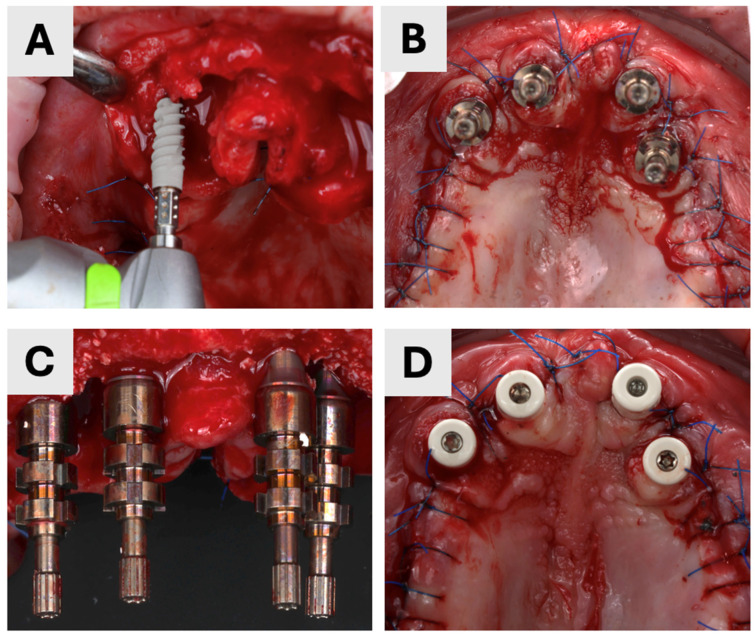
Implant placement and abutment connection following bilateral sinus lift augmentation. Due to the need for a healing period in the posterior maxilla, implants were placed only in the anterior region at sites 13, 11, 21, and 23 (FDI Notation System). (**A**) Insertion of implants into the anterior maxilla. (**B**) Occlusal view of the four implants placed and primary wound closure with non-resorptive sutures (Seralon blu 4-0, Serag-Wiessner GmbH & Co. KG, Naila, Germany). (**C**) Frontal view showing implant carriers in place prior to impression taking. (**D**) Final view of the healing abutments in place following suturing.

Due to the limited bone volume in the posterior region and the time required to achieve optimal tissue regeneration, implant placement in the molar area was not planned. Instead, four implants (Straumann^®^ BLX, Basel, Switzerland) were placed in the anterior maxilla at positions 13, 11, 21, and 23 (FDI Notation System), with the following dimensions:

- position 13: 3.75 × 10 mm;
- position 11: 3.75 × 8 mm;
- position 21: 3.75 × 8 mm;
- position 23: 4.0 × 10 mm (Figure 5).

Postoperative care included the application of cold compresses to both cheeks for the first 12 h following surgery to minimize swelling. The patient was advised to adhere to a soft, minced diet for two weeks to avoid mechanical trauma to the surgical sites. Systemic antibiotic prophylaxis consisted of sulfamethoxazole/trimethoprim (NOPIL^®^ forte 800/160 mg, Mepha Pharma AG, Switzerland), administered orally twice daily for five days, beginning 24 h prior to surgery. To support gut microbiota during antibiotic therapy, a probiotic supplement (OMNi-BiOTiC^®^ 10, Institut AllergoSan Pharma GmbH, Graz, Austria) was prescribed at a dosage of two daily doses for the first five days starting 24 h preoperatively, followed by once-daily administration for an additional 14 days. This regimen was selected in accordance with Swiss antimicrobial stewardship practices favoring narrower-spectrum agents or alternatives in patients intolerant to β-lactam antibiotics. No adverse events or postoperative infections were reported. Analgesic therapy included oral administration of paracetamol (MEPHADOLOR^®^ Neo 500 mg, Mepha Pharma AG), with one tablet taken one hour postoperatively, a second tablet at five hours, and a third tablet at nine hours. Local oral care involved the application of antiseptic gel (Blue^®^m Oral Gel, Zwolle, The Netherlands) to the surgical sites twice daily for 14 days using minimal pressure. Beginning 24 h postoperatively, the patient was instructed to gently rinse the mouth three times daily with antiseptic mouthwash (Blue^®^m Mouthwash, Zwolle, The Netherlands) and to perform atraumatic mechanical plaque control using a soft-bristled surgical toothbrush.

**Figure 6:**
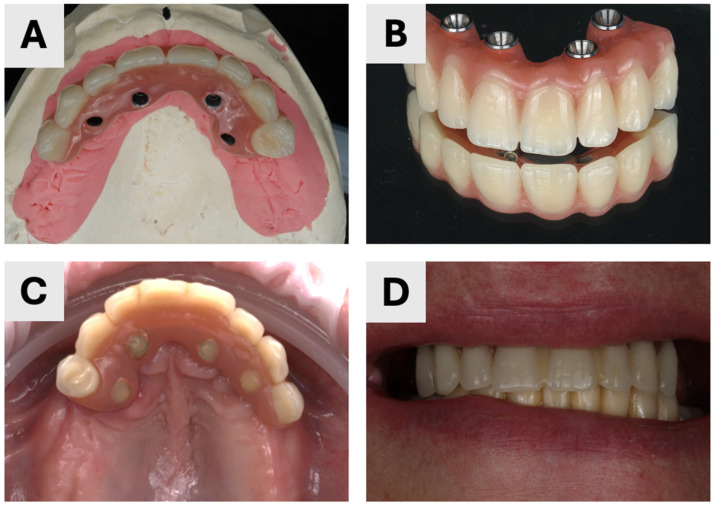
Provisional prosthetic rehabilitation following implant placement. (**A**) Laboratory view of the screw-retained temporary prosthesis on the working model. (**B**) Occlusal and frontal view of the finalized provisional restoration prior to delivery. (**C**) Intraoral try-in of the provisional prosthesis, occlusal view. (**D**) Frontal view of the patient in full intercuspidation with the provisional prosthesis in place.

An impression was taken during the same appointment, and a provisional fixed prosthesis was delivered within a few hours. Occlusion was evaluated both statically and dynamically using digital tools while the laboratory technician finalized the temporary restoration (Figure 6).

### 2.3. Reentry and Implant Placement Bilaterally at the Augmented Maxillary Sinus Site

**Figure 7:**
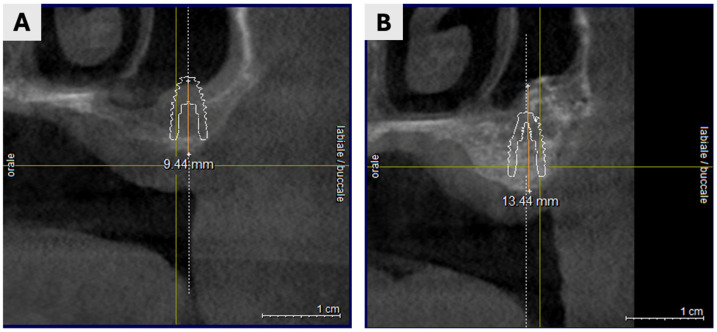
CBCT evaluation of vertical bone height six months after bilateral sinus floor elevation, prior to implant placement. (**A**) Left maxillary sinus showed vertical bone height of 9.44 mm. (**B**) Right maxillary sinus showed vertical bone height of 13.44 mm. Both sites demonstrated sufficient vertical bone volume for implant placement, with an intact Schneiderian membrane and no radiographic signs of pathology.

After a healing period of six months, a reentry procedure was performed to assess the previously augmented maxillary sinus sites bilaterally. Prior to reentry, a CBCT scan was obtained to confirm the dimensional stability of the augmented areas and to plan an implant placement. Radiological evaluation demonstrated vertical bone formation in both sinuses. In the left sinus, treated with a graft-free, magnesium membrane stabilized approach, residual bone height increased from 1.01 mm to 9.44 mm, corresponding to a vertical bone gain of 8.43 mm. In the right sinus, treated with a conventional lateral sinus lift, residual bone height increased from 3.00 mm to 13.44 mm, resulting in a vertical bone gain of 10.44 mm. The newly formed bone exhibited a homogeneous radiographic appearance, and the elevated Schneiderian membrane remained intact, with no signs of pathological changes or sinus complications. Implant positions were planned based on the newly established bone levels (Figure 7).

**Figure 8:**
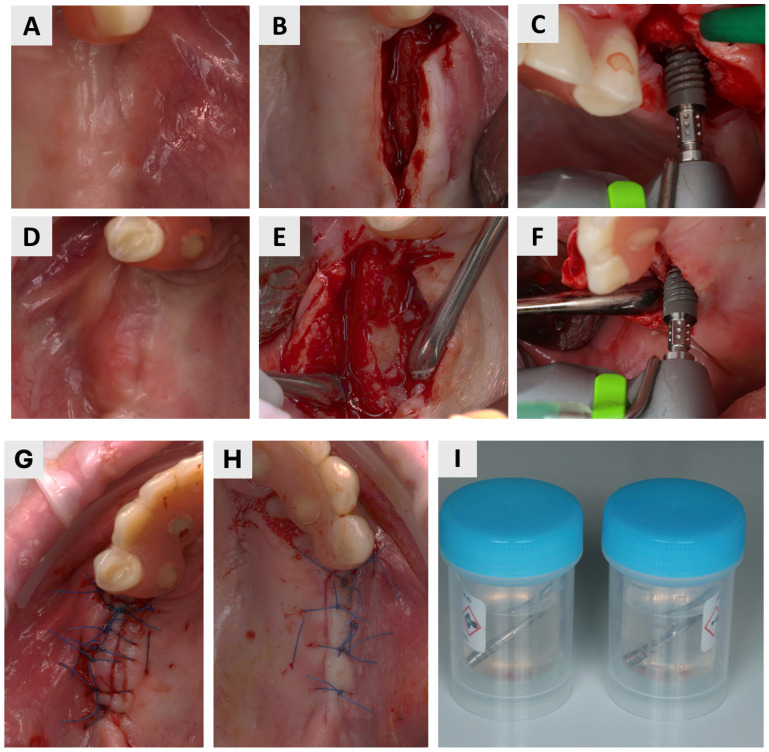
Surgical reentry, implant placement, and bone core biopsy harvesting after maxillary sinus augmentation. (**A**) Clinical view showing healthy soft tissue contours on the left (graft-free) side, six months post-surgery. (**B**) Full-thickness mucoperiosteal flap elevation to expose the underlying alveolar ridge. (**C**) Implant placement at site 26 following standard drilling protocol. (**D**–**F**) Identical clinical procedure performed on the contralateral (right) side, including flap elevation and implant placement at site 16. (**G**,**H**) Primary wound closure achieved bilaterally with tension-free suturing. (**I**) Bone core biopsy samples harvested from both sites (left and right) and fixed in 4% paraformaldehyde for histological analysis.

Dental implants were placed bilaterally. Implants with dimensions of 4.5 × 8 mm (W-body) were inserted at sites 16 and 26, corresponding to the regions of the maxillary first molars. In addition to implant placement, the bone core biopsies were obtained from the central portion of the pre-existing defect on the left and right side, directly beneath the implant site, from the occlusal point of view. The samples were immediately fixed in 4% paraformaldehyde solution and submitted for histological analysis. The histological evaluation was performed using standard processing and staining procedures, as detailed in the following section (Figure 8).

### 2.4. Final Prosthetic Phase and Restoration Delivery

**Figure 9:**
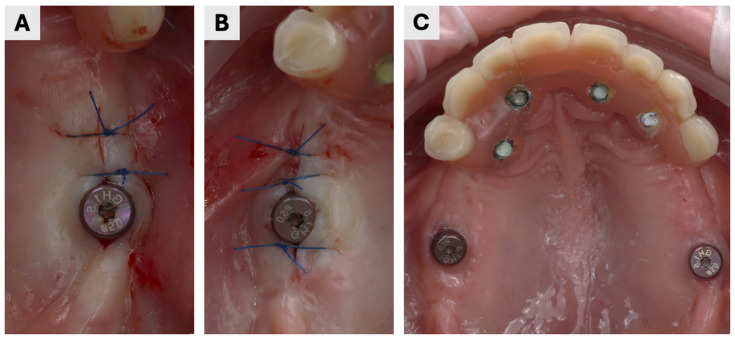
Placement of healing abutments following uncovering of the implants. (**A**) Clinical view of the left posterior maxilla after insertion of the healing abutment at site 26 and soft tissue suturing. (**B**) Corresponding view of the right side after healing abutment placement at site 16. (**C**) Postoperative occlusal view after suture removal, showing excellent soft tissue healing around posterior implants.

After a healing period of 15 weeks, a second-stage surgery was performed to expose the dental implants to enable the connection of healing abutments and prepare the site for the final restoration (Figure 9).

**Figure 10:**
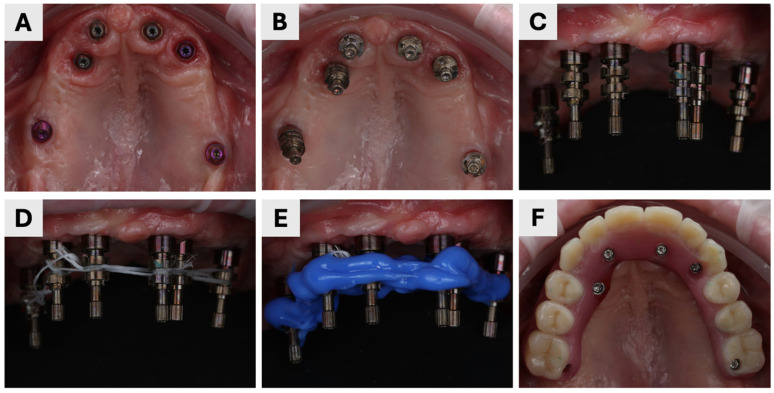
Analog impression procedure and passive fit verification for the definitive prosthetic rehabilitation. (**A**) Occlusal view of the implant sites following removal of healing abutments; small protective covers are visible inside the implants. (**B**) Occlusal view of transfer copings (impression posts) placed at all six implant sites. (**C**) Frontal view showing correct seating and alignment of the transfer copings. (**D**) Splinting of the transfer copings using LuxaBite, bis-acryl based material, known for its hardness and stability and low-shrinkage bite registration material to ensure positional accuracy. (**E**) Final analog impression taken using 3M Permadyne is made of polyether, a type of material known for its dimensional stability and accuracy in impressions. (**F**) Occlusal view of the try-in restoration with teeth set in wax on a laser-sintered titanium framework, used for esthetic and functional verification prior to finalization.

Then, the definitive impressions were taken. Due to the full-arch nature of the case, an analog impression technique was selected, as it remains the gold standard for achieving high accuracy and passive fit in such restorations. Transfer abutments were connected and splinted using dental floss and a low-contraction bite registration material to ensure dimensional stability. Maxillomandibular records were taken, and the occlusion was registered. A laser-sintered titanium framework was fabricated by the dental technician and tested to assess the fit. The passive fit was confirmed clinically (Figure 10).

**Figure 11:**
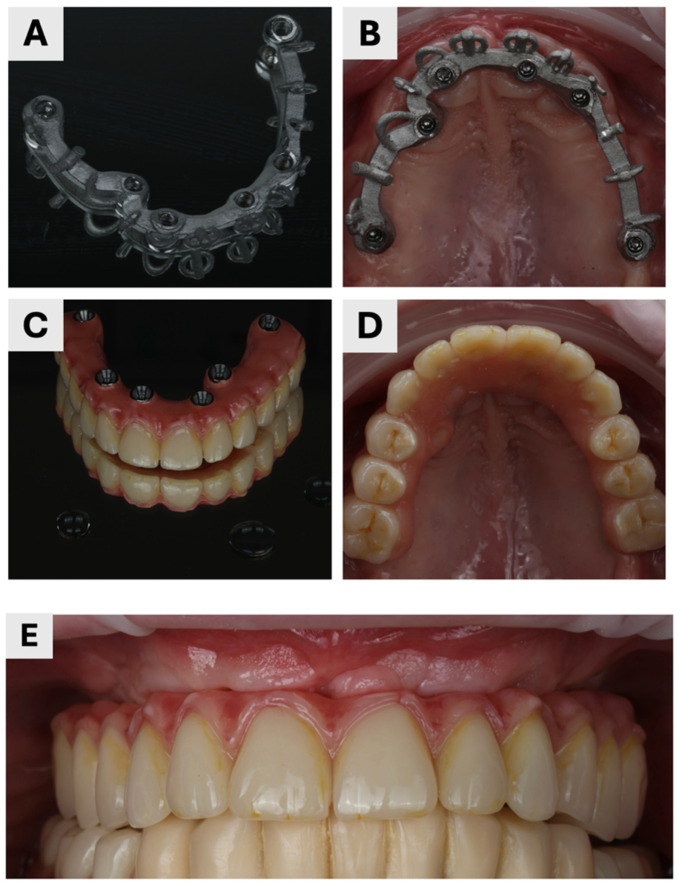
Definitive full-arch prosthetic restoration supported by six implants in the maxilla. (**A**) Laser-sintered titanium framework fabricated in the dental laboratory. (**B**) Clinical try-in of the titanium framework to verify passive fit in the patient’s mouth. (**C**) Laboratory view of the finalized full-arch screw-retained prosthesis with pink composite gingival mimicry and ceramic layering. (**D**) Occlusal view following intraoral delivery of the definitive prosthesis with access channels sealed with composite. (**E**) Frontal view of the final restoration immediately after screw-tightening, demonstrating esthetic integration and harmonious gingival contours.

Following the esthetic try-in phase, the definitive full-arch screw-retained prosthesis was delivered. The final restoration was torqued to the manufacturer-recommended values. The screw access channels were sealed using pink composite resin in areas corresponding to soft tissue (for esthetic integration) and white composite stained with brown pigment to mimic natural occlusal anatomy in functional zones (Figure 11).

### 2.5. Histological Evaluation

The retrospective histological analysis was performed at the Faculty of Dental Medicine and Health, Osijek, Croatia. Trephine biopsies were fixed in 4% formaldehyde for two weeks and subsequently decalcified in ethylenediaminetetraacetic acid (Decalcifier soft^®^, Solvagreen^®^, Karlsruhe, Austria). Following decalcification, samples were processed using a tissue processor (MTP, SLEE Medical GmbH, Mainz, Germany), embedded in paraffin wax (MPS/P, SLEE Medical GmbH, Mainz, Germany), and sectioned at a thickness of 3 µm using a microtome (CUT 4062, SLEE Medical GmbH, Mainz, Germany). The sections were stained with hematoxylin and eosin (H&E) and examined under a light microscope (Leica Microsystems GmbH, Wetzlar, Germany) equipped with a Zeiss Axio Imager M2 digital camera (Carl Zeiss, Oberkochen, Germany). Histological evaluation was performed by a single examiner not involved in the surgical procedures and without any affiliation to the biomaterial manufacturer (M.Č.).

Digital microphotographs were analyzed using ImageJ software (v 1.54, NIH, Bethesda, MD, USA) for histomorphometric quantification. A single representative section from the central portion of each biopsy was used for analysis. The region of interest (ROI) was defined to encompass the regenerated compartment of the sinus augmentation site, excluding the pre-existing native bone at the crestal margin and any peripheral artifacts. The total area (TA), newly formed bone (BT), and bone substitute (BS) were measured, while the connective tissue (CT) fraction was calculated as TA − (BT + BS). Values were expressed as volume percentages (%).

**Figure 12:**
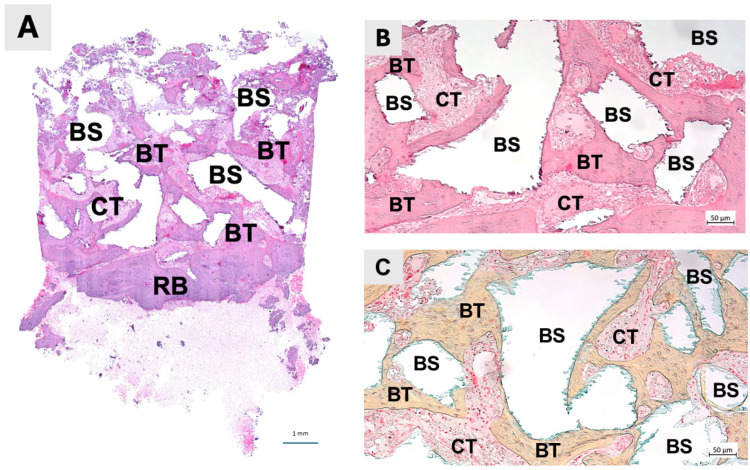
Histological analysis of the specimen harvested from the right maxillary sinus, where conventional lateral sinus augmentation with xenograft was performed. (**A**) Overview of the entire histological section stained with hematoxylin and eosin, illustrating the presence of newly formed bone (BT), connective tissue (CT), and residual bone (RB). RB is a common finding in sinus lift biopsies due to the nature of trephine harvesting, which includes a portion of the native crestal bone. Bone substitute (BS) particles are observed as well, integrated within the newly formed tissue. (**B**) Higher magnification of the region of interest showing intimate contact between BS and BT, along with interspersed CT (Hematoxlyin-eosin staining A,B). (**C**) Masson-Goldner trichrome staining reveals good bony maturation. Integration of the bone substitute granules into the newly formed bone tissue. No signs of active inflammation or foreign body reaction are visible.

**Figure 13:**
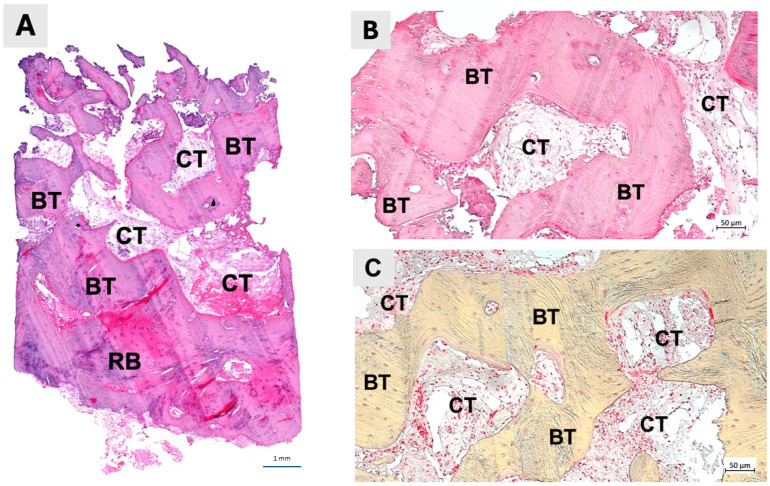
Histological analysis of the specimen harvested from the left maxillary sinus, where a graft-free sinus lift was performed using a resorbable magnesium membrane. (**A**) Low-magnification overview of the specimen stained with hematoxylin and eosin, showing a dense distribution of BT and CT, along with residual bone. No BS particles are visible, consistent with the graft-free nature of the procedure. (**B**) Higher magnification of the central region suggests a well-organized lamellar bone structure with mature BT and interspersed CT (Hematoxylin-eosin staining A and B). (**C**) Masson-Goldner trichrome staining shows a good bony implantation bed for implant insertion and newly formed trabecular bone tissue with active osteoblasts. The absence of inflammatory cells or foreign body reaction and the presence of embedded osteocytes support advanced bone maturation.

Both specimens demonstrated the presence of BT with CT. In the specimen harvested from the right maxillary sinus, where the conventional lateral sinus lift with xenograft was performed, bovine-derived BS particles were observed, well-integrated within the surrounding bone matrix. Evidence of active bone regeneration was clearly visible in both samples, particularly in areas rich in osteoblasts. Mature osteocytes embedded within lacunae were also identified, confirming ongoing bone maturation. Importantly, no signs of inflammation or foreign body reaction were detected in either specimen (Figure 12 and Figure 13).

Histomorphometric analysis revealed a higher percentage of BT in the specimen obtained from the left side, where the graft-free sinus lift using a magnesium membrane was performed (Table 1).

### 2.6. Radiological Assessment Two Years Following the Procedure

**Figure 14:**
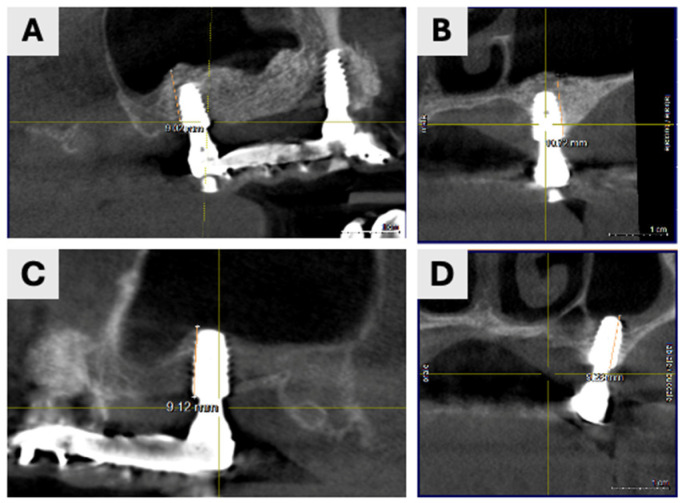
Radiological assessment at the two-year follow-up. (**A**) Sagittal CBCT view of implant site 16, demonstrating a vertical bone height of 9.02 mm with persistent radiopaque graft material surrounding the implant apex. (**B**) Coronal CBCT view of the same implant site (16), showing a vertical bone height of 10.72 mm. (**C**) Sagittal CBCT view of implant site 26, demonstrating a vertical bone height of 9.12 mm and mature native bone surrounding the implant. (**D**) Coronal CBCT view of the same site (26), showing a vertical bone height of 9.28 mm, with stable peri-implant bone and no radiographic signs of pathology or radiolucency.

A CBCT scan was performed two years after bilateral sinus augmentation to evaluate the long-term dimensional stability and bone remodeling at both sites. Radiological assessment demonstrated stable peri-implant bone conditions in both maxillary sinuses, with satisfactory vertical bone height and no signs of sinus pathology or implant-related complications. On the right side, which was conventionally treated, the vertical bone height ranged from approximately 9.02 to 10.72 mm at the two-year follow-up. Radiographic evaluation revealed the persistent presence of radiopaque graft particles within the augmented area (Figure 14A,B). On the graft-free sinus lift side, the vertical bone height measured approximately 9.12–9.28 mm, suggesting minimal dimensional change compared with the six-month evaluation. The augmented area exhibited homogeneous radiodensity and a trabecular pattern consistent with mature, newly formed native bone surrounding the implant (Figure 14A,B).

Comparative analysis between baseline and the two-year follow-up demonstrated vertical bone gain on both sides. The conventionally augmented sinus increased from 3.00 mm to 10.72 mm (+7.72 mm), whereas the graft-free magnesium membrane site increased from 1.01 mm to 9.28 mm (+8.27 mm).

## 3. Discussion

This bilateral case report presents a clinical, radiographic, and histological comparison between two maxillary sinus augmentation techniques performed in the same patient. For that purpose, a conventional lateral sinus lift using xenogeneic bone graft and collagen membrane, and a graft-free sinus lift using a resorbable magnesium membrane were done. Both approaches resulted in successful implant placement after a six-month healing period. However, histomorphometric analysis revealed a higher percentage of newly formed bone in the site treated with the graft-free technique (22.53% vs. 56.43%). Radiographic follow-up two years postoperatively suggested stable osseointegration of both implants, with native bone visible around the graft-free site and residual graft particles still present on the grafted side. In the following discussion, the results are interpreted in the context of existing literature.

The handling characteristics of the resorbable magnesium membrane have been described in various clinical applications, including the magnesium membrane shield technique introduced by Elad et al. [20]. In the present case, the membrane demonstrated similar properties, especially its form stability and ease of insertion through the limited crestal access. This mechanical stability allowed the membrane to be positioned securely without the need for additional fixation devices. In conventional sinus lift procedures, the use of membrane fixation, typically with titanium tacks or pins, is often required to prevent membrane displacement during healing. These fixation systems are frequently used when membranes lack inherent rigidity or when large sinus perforations necessitate mechanical stabilization [23,24]. In contrast, the magnesium membrane used in this report achieved stable positioning without the use of pins or tacks, therefore reducing surgical complexity and time. This fixation-free approach not only simplifies the operative protocol but may also reduce patient morbidity by eliminating the need for additional hardware in the surgical site.

The present case demonstrated successful bone regeneration as evidenced by histological and histomorphometric analysis performed six months after sinus augmentation. On the side treated with the resorbable magnesium membrane and no bone graft, histological evaluation revealed a well-organized trabecular structure, with the presence of BT interspersed with CT. Active osteoblasts were observed along bone surfaces, and mature osteocytes embedded within lacunae suggested bone maturation. No signs of inflammation or foreign body reaction were present. Importantly, the membrane-treated site exhibited a higher percentage of BT compared to the contralateral, grafted sinus. This suggests the regenerative potential of a graft-free sinus lift approach when supported by stable membrane-based compartmentalization. The form-stable magnesium membrane maintained the subantral space due to its intrinsic rigidity and shape memory, which allowed stable positioning without the need for fixation. In contrast to conventional collagen membranes, which often require pin or tack fixation because of their flexibility, the magnesium membrane provided mechanical support to preserve the elevated compartment. While magnesium membranes do not serve as long-term scaffolds due to their full resorption, their initial mechanical stability appears to play a crucial role in supporting natural healing [25]. This is also in accordance with the histomorphometric results (47% of newly formed bone) reported by Blašković et al. [26], who evaluated magnesium membrane in a different clinical indication and demonstrated successful bone regeneration in the absence of inflammation. This approach also aligns with the concept previously described in studies on crestal sinus lifts using barrier membranes alone, which describe the importance of space maintenance, clot protection, and undisturbed healing over graft material selection [27,28]. Taken together, the results of the current case report highlight the potential for simplified, biologically driven sinus augmentation protocols with reduced morbidity and surgical complexity.

At the two-year radiographic follow-up, both augmentation approaches demonstrated stable and clinically relevant vertical bone formation. The conventionally treated sinus showed an increase in residual bone height from 3.00 mm to 10.72 mm (+7.72 mm), while the graft-free site stabilized with a resorbable magnesium membrane increased from 1.01 mm to 9.28 mm (+8.27 mm). Although absolute bone height remained marginally higher on the conventional side, the overall vertical gain was slightly greater at the magnesium site, despite more compromised baseline conditions. Given the single-case, split-mouth design, these findings should be interpreted cautiously; however, they indicate that the magnesium membrane technique may provide comparable long-term regenerative potential to conventional grafting.

Magnesium salts and their degradation products have been studied for their role in bone regeneration. In the present case, the higher percentage of newly formed bone observed at the graft-free magnesium membrane site may be explained by the local release of magnesium ions during membrane degradation. These ions are known to stimulate osteoblast proliferation, improve alkaline phosphatase activity, and promote matrix mineralization. Those are all processes essential for early bone formation [19,29]. In addition, magnesium has been reported to modulate the local inflammatory response and support balanced bone remodeling [14], which may contribute to the homogeneous bone structure and radiological appearance observed at the two-year follow-up.

Experimental studies in critical-size defect models suggest that locally delivered magnesium ions can activate osteogenic signaling pathways, thereby improving both the quantity and quality of newly formed bone [30]. These findings are particularly relevant for degradable magnesium-based materials, such as the resorbable magnesium membrane used in the present case. As the membrane degrades, it gradually releases magnesium ions into the surrounding microenvironment, potentially contributing to a biologically favorable milieu for bone regeneration.

Although this split-mouth case report presents a novel application of a resorbable magnesium membrane in a graft-free sinus lift procedure, the findings must be interpreted within the context of certain limitations. Most importantly, the outcomes are derived from a single-patient design, which limits the generalizability of the results. Additionally, while a two-year clinical and radiographic follow-up provides valuable insight into the mid-term stability of the regenerated bone, longer observation periods are needed to fully assess the long-term performance of this approach. Also, a limitation of this case report is the absence of standardized densitometric analysis. Although radiographic density was considered, CBCT-based gray values were not used for quantitative assessment due to their limited reliability and lack of direct comparability with Hounsfield units. However, case reports that introduce a novel treatment approach, such as the one presented here, remain a crucial starting point for identifying innovative clinical strategies and generating hypotheses for future research.

Future research should validate these findings through larger studies. Such investigations will help confirm the reliability and long-term performance of the graft-free sinus lift concept using a resorbable magnesium membrane. Until such data become available, the encouraging histological, radiological, and clinical outcomes observed in this report support the biological and clinical benefits of using a resorbable magnesium membrane in graft-free sinus lift procedures.

## Figures and Tables

**Table 1:** Histomorphometrical results of the specimen analysis (6 months after augmentation).

Tissue	Right Sinus (%)	Left Sinus (%)
BT (Newly Formed Bone)	22.53	56.43
CT (Connective Tissue)	45.35	43.57
BS (Bone Substitute)	32.12	0.00

Right sinus = xenograft + collagen membrane; Left sinus = graft-free Mg membrane site. Values are expressed as percentages (%) of the total analyzed tissue area.

## Data Availability

The original contributions presented in this study are included in the article. Further inquiries can be directed to the corresponding author.
